# Social network-based group intervention to promote uptake of cervical cancer screening in Uganda: study protocol for a pilot randomized controlled trial

**DOI:** 10.1186/s40814-022-01211-z

**Published:** 2022-12-07

**Authors:** Rhoda K. Wanyenze, Joseph K. B. Matovu, Kathryn Bouskill, Margrethe Juncker, Eve Namisango, Sylvia Nakami, Jolly Beyeza-Kashesya, Emmanuel Luyirika, Glenn J. Wagner

**Affiliations:** 1 School of Public Health, Makerere University, Kampala, 7072 Uganda; 2 Busitema University Faculty of Health Sciences, Mbale, Uganda; 3 RAND Corporation, Santa Monica, CA USA; 4 Rays of Hope Hospice Jinja, Jinja, Uganda; 5 African Palliative Care Association, Kampala, Uganda; 6 Mulago Specialized Women and Neonatal Hospital, Kampala, Uganda; 7 School of Medicine, Makerere University, Kampala, Uganda

**Keywords:** Cervical cancer, Screening uptake, Social-network intervention

## Abstract

**Introduction:**

Cervical cancer (CC) is the most common cancer and accounts for one quarter of all cancer-related deaths among women in Uganda, where lifetime CC screening is estimated to be as low as 5%. This study will evaluate the feasibility, acceptability, and preliminary efficacy of a social network-based group intervention designed to empower women who have received CC screening to encourage women in their social network to also screen.

**Methods:**

Forty adult women (index participants) who have recently screened for CC will be recruited, 20 of whom will be randomly assigned to take part in the intervention and 20 to the wait-list control. Each index participant will be asked to recruit up to three female social network members (i.e., alters; maximum total = 120 alters) who have not screened for CC to participate in the study. Assessments (survey and chart abstraction) will be administered at baseline and month 6 to index and alter participants. The primary outcome is CC screening among participating alters, with a secondary outcome being engagement in CC prevention advocacy among index participants. Repeated-measure multivariable regression analyses will be conducted to compare outcomes between the intervention and control arms.

**Discussion:**

If successful, this intervention model has the potential not only to impact uptake of CC screening and treatment but also to establish a paradigm that can be applied to other health conditions.

**Trial registration:**

NIH Clinical Trial Registry NCT04960748 (clinicaltrials.gov).

## Introduction

Cervical cancer (CC) is the most common cancer and accounts for ~25% of all cancer-related deaths among women in Uganda, who have one of the highest incidence rates in the world at 54.8 per 100,000 [1–3]. CC screening via visual inspection of cervix with acetic acid (VIA), and thermal therapy for precancerous lesions, is available for free or a low cost in Uganda, while radiotherapy is prescribed for advanced disease but sub-optimally available due to high patient load and still too costly for most women to access, despite the subsidies. This highlights the importance of screening to prevent onset of cancerous lesions, yet most (80%) Ugandan women have advanced CC disease (stage 3 or higher) at initial presentation for care [4], and it is estimated that just 5% of women have ever screened for CC [4–6].

A key structural barrier to uptake of CC screening has been poor access [7], but national government initiatives are underway to ramp up availability, especially screening and early-stage treatment [8]. Nonstructural barriers to screening include lack of awareness and misinformation, embarrassment with the screening procedure (especially if the provider is male), and fear of screening results [6, 7, 9, 10]. Stigma associated with CC being a sexually transmitted infection, and the degrading symptoms of advanced disease (e.g., offensive discharge, heavy bleeding, fistulas, incontinence) that often lead women to be isolated and feel ashamed [6, 7, 9, 10], may be impediments to treatment more so than screening. Facilitators to screening include outreach services, support and encouragement from others, and knowing someone who has been screened or diagnosed [6, 7]; however, no studies have tried to leverage and diffuse information through social networks to improve uptake of screening or treatment [7].

One promising approach is to empower women who have personal experience with being screened for CC, and treated if applicable, to act as advocates, and to encourage other women they know to get screened. Building on theories of social diffusion [11], cognitive consistency [12], and social influence [13], which posit that behavior change can be initiated by a few and diffused to others through modeling, advocacy, and shifts in social norms, peer advocacy interventions have been shown to promote prevention and screening, raise awareness, and reduce stigma in the context of HIV [14–16]. In the context of CC, peer education has been evaluated at the level of healthcare workers [17], male partners [18], and women at risk [10], but not through social networks. We recently developed and tested a network-based advocacy group intervention, *Game Changers*, that mobilized people living with HIV in Uganda to act as change agents for HIV prevention within their social networks [19]. The intervention, which targeted reduction of internalized stigma, disclosure skills, healthy living, and advocacy skills building, resulted in reduced HIV stigma, increased HIV disclosure and engagement in advocacy among the participants, and increased HIV testing and condom use among their network members [19].

**Fig. 1:**
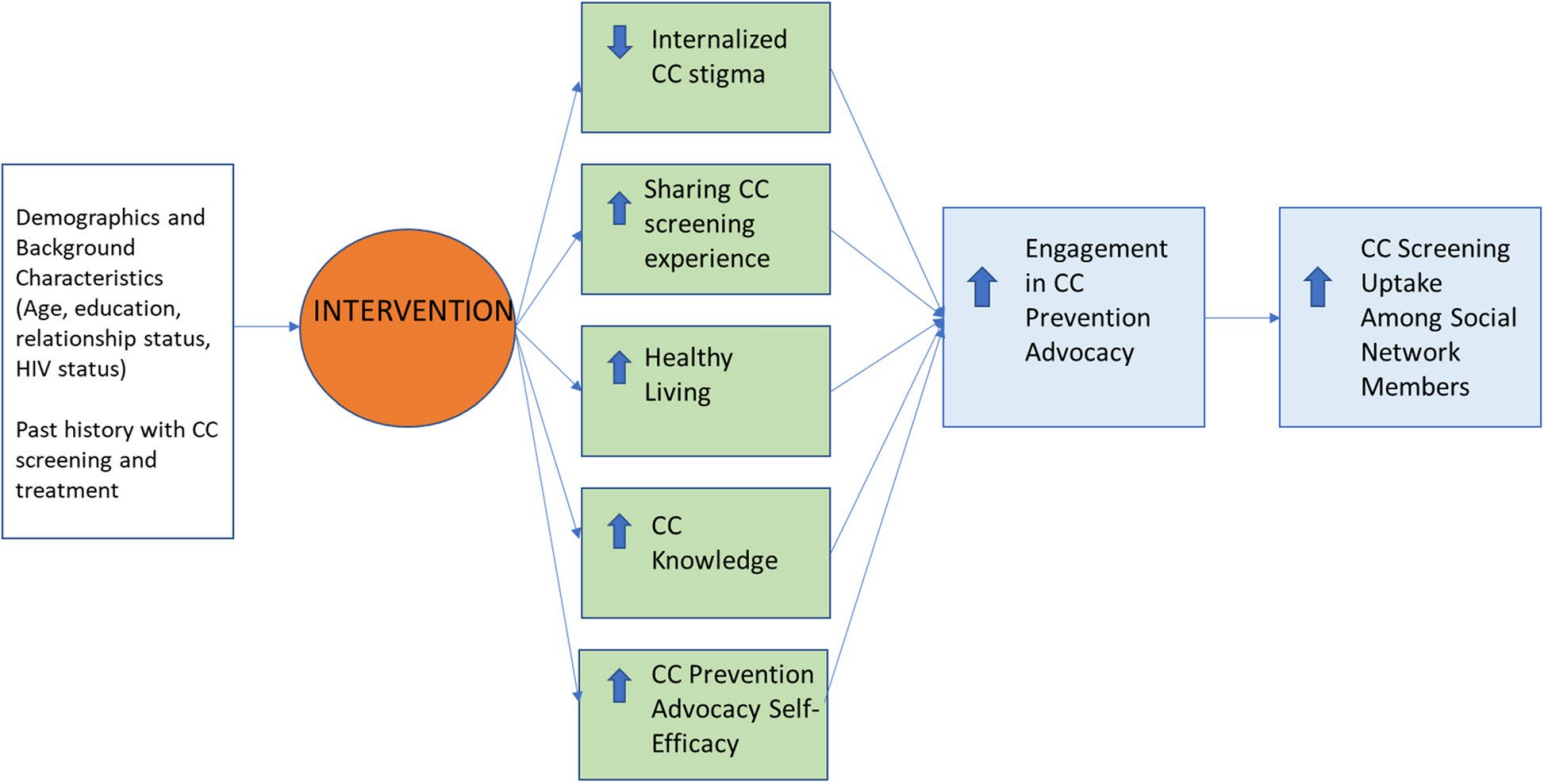
Conceptual framework for promotion of cervical cancer (CC) prevention advocacy among screened women to affect CC screening among social network members

We believe these same processes are relevant to empowering women who have screened for CC to act as change agents for CC prevention and treatment. We believe that effective advocacy first requires coping with internal and external stigma, particularly if screening resulted in evidence of infection and need for treatment, and achieving a level of self-acceptance. Self-acceptance facilitates comfort with sharing one’s screening experience with others and being able to receive support from others. Sharing one’s experience of screening raises the credibility of one’s advocacy for CC screening and treatment and better enables successful encouragement of others to seek screening and treatment (if needed). However, disclosure of CC risk can have both positive (increased support) and negative (rejection, ridicule, shame) outcomes, so disclosure of decision-making skills is important. To be effective advocates, women must model the behaviors they encourage others to adopt, by prioritizing healthy living including adherence to CC rescreening schedules. Lastly, learning communication skills and strategies for when, how, and who to engage with advocacy, is key to effective advocacy. These processes make up the framework (Fig. 1) guiding the intervention we are testing in this study. It draws on existing evidence-based network-driven interventions in the context of HIV prevention [20–23] and mechanisms such as experience sharing and role playing to foster peer support.

We will conduct a randomized, controlled pilot of the intervention to evaluate the feasibility, acceptability, and preliminary efficacy of the intervention to increase CC screening among women in the social networks of the intervention recipients. The protocol for this trial is described in detail in this paper. If results are promising, this pilot study will inform a more robust evaluation of the intervention in a larger trial. This intervention model has the potential not only to impact uptake of CC screening and treatment but also to establish a paradigm that can be applied to other health conditions.

## Methods

### Study design

This study will pilot test a social network-based group intervention that aims to empower and mobilize women who have been screened for CC to advocate for CC screening and treatment in female members of their social network (referred to as “alters”). We will pilot the intervention in a randomized controlled trial of 40 women who have been screened for CC (referred to as index participants), with 20 randomly assigned to receive the intervention (in two groups of 10) and 20 to the wait-list control [(each evenly stratified by age (under and over age 35) and history of CC-related treatment)]. Individual randomization entails risks for contamination, but statistical models suggest that ≥ 30% of the control arm must receive the equivalent of a full-strength intervention to result in meaningful contamination [24]. We opted for the wait-list control rather than an attention control, because this provides clearer findings for policy makers when considering intervention effects, and adequate control for attention may not be feasible given the 2-h duration and group dynamics of the workshops. The control arm receives the intervention after the completion of month-6 follow-up assessments. There is no way to blind the participants on whether or not they receive the intervention; this could potentially influence the outcomes, as clients may feel more or less incentivized to perform well in light of whether or not they receive the intervention. We do not see a way to prevent this potential bias, nor a way to distinguish such effects from actual intervention effects, but this limitation will be cited in reports of study findings. Data will be collected at baseline and month 6 from index participants and up to three alters of each index participant (max. total = 120 alters) who have not previously screened for CC. The primary outcome is alter CC screening over the 6-month follow-up period; secondary outcomes are index participant reported engagement in CC screening advocacy, internalized CC stigma, and disclosure of CC screening experience.

#### Sample size

The small sample limits statistical power, but our goal is to assess the preliminary effect size and other parameters to inform a future larger trial. Assuming 120 alters enroll, attrition rate of 10% among alters (we have retained 90–94% over 12 months in past studies in Uganda) [25, 26], *ICC* = .01 to control for clustering within the alters of each participant, and a 5% rate of receiving CC screening in control alters, the effective sample size will be 53 per arm, enabling us to detect a medium effect size (Cohen’s *d* = 0.57; 19 percentage point difference in CC screening among alters during the 6-month follow-up period), with 80% power (alpha = .05). If ICC is as high as .05, and as few as 80 alters enroll, the detectable effect size = 0.72 (25.5 percentage point difference between groups).

### Setting

The study will take place in Namayingo, a rural community in the Busoga region of Uganda, and specifically Buyinja and Banda health centers. In addition to CC screening and thermal therapy that takes place at the two health centers, Rays of Hope Hospice Jinja (RHHJ) conducts 1- to 3-day CC screening “camps” in Namayingo, during which it provides free or low-cost VIA screening and thermal therapy, as well as referrals and assistance to access more invasive CC treatment. Women screened in the RHHJ camps are registered in a database used to track them and facilitate further follow-up and outreach. However, these camps did not take place in the study setting during the course of the 6-month follow-up period.

With support from the leadership and community volunteers of the Buyinja and Banda health centers, as well as RHHJ and African Palliative Care Association, we composed a community advisory board consisting of members of key stakeholder groups (district health officials, community leaders, reproductive health providers, and women living with CC risk). This board provided key input into the development and adaptation of the intervention and instrumentation and will hold half-day meetings at key junctures throughout the implementation of the trial to inform the need for any adjustments to the intervention, as well as interpretation and dissemination of findings.

### Participants

Women are eligible to enroll as index participants if they are age 18 years or older, have screened for CC within the past year, have stable health status (i.e., not in end stages of disease), and shared their CC screening experience with at least one alter who is perceived to not have screened for CC in the past 3 years. Alter participants are eligible if they are at least 18 years of age, are recruited by an index participant, and report never being screened for CC.

Index participants will be recruited through the RHHJ database of women who have received CC screening and referral from Buyinja and Banda providers who have screened women for CC. An RHHJ staff member or health center provider will inform eligible women of the study, and those who express interest in participating will be referred to the study coordinator for formal eligibility screening and implementation of consent procedures. Women who provide written informed consent will then be administered the baseline assessment and be randomly assigned to the intervention or control arm. To recruit alters, we will use data collected from the baseline survey assessment of female social network members to randomly select 5 alters who know the participant’s CC screening experience (or as many as there are if < 5) and ask the participant if she is comfortable asking 3 of these alters to participate. The index participant will be asked to call each selected alter at the end of the interview to describe the study in the presence of the coordinator, who will schedule a study visit for alters who express interest in participating. If an alter refuses or cannot be reached, a replacement will be randomly selected from the list of alters who know the index participant’s CC screening experience.

### Intervention

Using focus groups of women with and without CC screening experience, and a focus group of male partners, we collected formative data to adapt the *Game Changer* intervention for the context of CC. A key difference revealed by these data between the HIV context and that of CC is that internalized stigma did not appear to be an impediment to CC screening per se, but rather stigma, fears, and worries acted more as barriers to biopsies and treatment, and to sharing one’s screening result, once a woman learned that she was positive for the sexually transmitted human papillomavirus and at risk for CC. Support from male partners for screening and treatment was also cited as important, and men who had some basic level of knowledge about CC seemed largely supportive of women being screened.

The adapted intervention consists of 7 workshops. *Workshop 1* focuses on *addressing fears and concerns related to CC risk and use of self-compassion and peer support to overcome these fears*, as well as introducing the overall vision for empowering women to become change agents for CC prevention and treatment. With participants feeling more supported, *Workshop 2* focuses on *building skills and decision-making for sharing one’s personal CC screening experience*, knowing to whom to disclose and when and how to initiate and navigate disclosure and conversations about CC. *Workshop 3* builds skills and motivation for *healthy living* so that their own behavior (e.g., good nutrition, periodic VIA screens) is consistent with the behavior they encourage in others, as well as learning facts and myths related to CC to facilitate accurate CC screening advocacy. *Workshop 4* introduces the concept of a social network and how one’s network can serve as a tool for CC prevention advocacy and dissemination of CC-related information. *Workshops 5 and 6* focus on the skills needed for successful *CC-related advocacy*, including strategies for how to start conversations about CC and to keep these discussions going, as well as learning effective communication skills (e.g., reflective listening, paraphrasing, open-ended questions). *Workshop 7* inspires a *commitment to ongoing CC advocacy* through peer solidarity and support. The workshops will be conducted using a structured facilitator manual, in the predominant local languages of Samia and Lusoga, by two trained women from Namayingo who have experience being screened for CC. Using principles of compassion-focused therapy [27], the workshops will be administered in a group format to facilitate the use of *sharing of experiences* to build support, solidarity, and motivation among participants; *group problem-solving* and *role playing* to build skills and self-efficacy; *setting personal goals* regarding healthy living, disclosure, and advocacy; and homework to reinforce practice of new skills and generate personal experiences to be processed in the workshops. The seven workshops will be conducted weekly over 2 months, and each workshop will last 2 h. Participants will receive 30,000 Uganda shillings (~US $8) for attending each workshop to cover transport costs.

#### Facilitator training, supervision, and fidelity monitoring

The facilitators will be trained by the senior investigators over 3 days. The training will include reviewing the manual, objectives for each workshop, step-by-step scripts and the key points to emphasize, and role playing and mock implementation of core exercises. Training will cover group facilitation, building rapport with and among participants, reflective listening, and dealing with group conflict. The supervisor of the facilitators will observe the implementation of each of the workshops to provide feedback and further training as needed during weekly supervision. To monitor implementation fidelity, the supervisor will complete a rating form after each session to record if objectives were met, exercises completed, level of participant engagement, difficulties encountered, and areas to improve.

### Measures

Assessments will be administered at baseline and 6-month follow-up, in the local languages, either Samia or Lusoga, depending on the preference of the participant. Assessments include a standard survey (index and alter participants) and social network assessment (index participant only); the assessment of the index participant will last about 60 min while just 20 min for the alter. The assessment will be administered using Network Canvas computer-assisted software. The constructs being measured are listed in Table 1; measures will be translated into Samia and Lusoga using standard translation/backtranslation methodology. CC screening and treatment utilization will be verified with abstracted medical chart data. Participants will receive 30,000 Uganda shillings (~US $8) for each completed assessment to cover transport costs.

**Table 1:** Constructs to be assessed

Constructs	Instrument
***Primary outcome***	
CC screening: index and alter	Self-report; chart abstraction
***Secondary outcomes***	
Receipt of CC-related treatment (e.g., thermal therapy; chemotherapy; radiation), if warranted: index and alter	Self-report; chart abstraction
Healthy living behaviors (condom use, diet, alcohol use, CC rescreening): index and alter	Developed in-house
Engagement/receipt of CC prevention and treatment advocacy: index and alter	Developed in-house
***Potential mediators***	
Internalized CC stigma: index	Kalichman et al. [28] adapted
Disclosure of CC screening and treatment results: index and alter	Developed in-house
Attitudes towards women with CC: index and alter	Developed in-house
Self-efficacy (adherence, disclosure, advocacy): index and alter	Chesney [29] adapted
CC-related knowledge: index and alter	Developed in-house
***Covariates/potential moderators***	
Demographics (age, education, work status, income, relationship status): index and alter	Developed in-house
Social support: index and alter	Moser et al. [30] adapted
Enacted CC stigma/discrimination: alter	Berger et al. [31] adapted

#### Social network assessment

Using Network Canvas software, each index participant will list 12 female adult alters (which is adequate to capture structural and compositional variability) [32] with whom they interact most. For each alter, we will gather information to assess *network composition* (e.g., age, HIV status, relation to index; perceived history with CC screening and treatment; knowledge of index’s CC screening and treatment). Index participants will report how well each alter knows each other alter to assess *network structure* (e.g., density or connectedness among alters). *To assess CC advocacy*, we will ask the index participant if they have encouraged the alter regarding CC screening and/or treatment, risk reduction behaviors (condom use), and any perceived resulting action (e.g., alter sought VIA screening). At follow-up, we will determine whether listed alters are the same or unique from those listed at baseline, which allows for both sequential cross-sectional analyses and longitudinal analyses of alter data. Our prior research shows that alter behaviors such as diagnosis disclosure and health-seeking behaviors can be accurately reported by index participants [33].

### Statistical analysis

To assess feasibility and acceptability, we will examine recruitment (percent of screened women and social network members who are approached to participate, decide to enroll), retention, workshop attendance, and participant feedback elicited in the process measures. To assess intervention effects on alter CC screening, *w*e will use an intent-to-treat approach. In addition to comparing the arms at each time interval (baseline, month 6), we will apply logistic generalized mixed models to our repeated-measure data to examine intervention effects, using an indicator for study arm, and time by arm interaction to indicate whether change differs between the arms. We will use imputation for item nonresponse and attrition weights to account for nonrandom dropouts using logistic regressions; if dropout is random, analyses will incorporate design effects. We will explore the sensitivity of significance levels and conclusions to a range of plausible ICC values (.005 to .05) for the outcomes. We will control for and examine interaction effects with intervention process variables (e.g., number of workshops completed) and characteristics of the index participant (e.g., age), as well as the alter (e.g., position in the network, knowledge of index participant’s CC risk).

We will explore factors that may mediate the intervention effects on the primary outcomes among variables targeted by the intervention*,* such as internalized CC stigma, disclosure (percentage of alters disclosed to), and engagement in CC screening advocacy. The sample size may be too small to do mediation analysis, but we will examine how potential mediators are associated with the primary outcomes and treatment condition using bivariate statistics.

### Ethics and dissemination

The study protocol has been reviewed and approved by the Makerere University School of Public Health Research and Ethics Committee (SPH-REC), College of Health and Sciences, Uganda, and cleared by the Uganda National Council of Science and Technology as per national research regulations. Any protocol modifications will be submitted to the SPH-REC for review, and participants will be informed if warranted. The trial is registered with the NIH clinical trial registry (clinicaltrials.gov) and assigned the number NCT04960748 (registration date: June 25, 2021). To ensure and maintain the scientific integrity of this human subject research project, and to protect the safety of its research participants, we will have a single, independent monitor (a reproductive health research scientist at the Makerere School of Public Health) to intermittently monitor study results and adverse event data (at months 3 and 6 of implementation of the intervention). The monitor will be provided with periodic reports which include subject enrollment, subject retention, reasons for dropping out, and a listing of all adverse events that are plausibly related to the intervention or study procedures. Adverse events that are considered directly related to the intervention or other aspect of study participation will be reported immediately to the monitor, the IRBs, and NIH. After review of the periodic reports, the monitor will make a recommendation regarding the continuation, modification, or termination of the study. All communications from the independent monitor will be shared with the IRBs and NIH.

As a first step for dissemination, reporting results will be documented on ClinicalTrials.gov in accordance with NIH requirements on dissemination of clinical trial results. Information submitted will occur no later than 12 months after the primary completion date. Results produced by this investigation will be presented at international conferences and published in a timely fashion, ideally in the last year of the study period. All final peer-reviewed manuscripts that arise from this proposal will be submitted to the digital archive PubMed Central for open access. De-identified data, assessment and intervention materials, and analytic code will be made available upon request from external researchers and following review and approval of the study team.

## Discussion

This study will conduct a randomized, controlled pilot evaluation of a social network-based group intervention designed to empower women who have screened for CC to advocate for CC screening and early treatment among women in their social networks. To our knowledge, no published study has used a network-driven approach and empowerment of screened women (some of whom have also been treated for CC risk) as change agents to increase CC screening and treatment, let alone in Africa. This is one of the few network-driven interventions to use social network data not only to evaluate effects on the behavior of network members but also to inform intervention strategies for targeting advocacy to bridging and popular network members, which may optimize the knowledge and support transfer for CC protective behaviors throughout a network. With essentially every family affected by CC risk in high-prevalence settings like Uganda, the intervention can dramatically impact the mortality and morbidity of CC through widely disseminated and targeted advocacy. This study will inform a future, larger controlled intervention trial, including a more extensive evaluation of effects on network members and the community. If successful, this intervention model has the potential to not only impact uptake of CC screening and treatment but also to establish a paradigm that can be applied to other health conditions.

## Data Availability

This is a protocol paper, and no data are available yet. De-identified data, assessment and intervention materials, and analytic code from this study will be made available with subsequent papers.
